# Left ventricular anatomy in obstructive hypertrophic cardiomyopathy: beyond basal septal hypertrophy

**DOI:** 10.1093/ehjci/jeac233

**Published:** 2022-11-28

**Authors:** Uxio Hermida, David Stojanovski, Betty Raman, Rina Ariga, Alistair A Young, Valentina Carapella, Gerry Carr-White, Elena Lukaschuk, Stefan K Piechnik, Christopher M Kramer, Milind Y Desai, William S Weintraub, Stefan Neubauer, Hugh Watkins, Pablo Lamata

**Affiliations:** School of Biomedical Engineering and Imaging Sciences, King’s College London, 5th Floor Becket House, Lambeth Palace Road, London SE1 7EU, UK; School of Biomedical Engineering and Imaging Sciences, King’s College London, 5th Floor Becket House, Lambeth Palace Road, London SE1 7EU, UK; Oxford Centre for Clinical Magnetic Resonance Research, Division of Cardiovascular Medicine, Radcliffe Department of Medicine, University of Oxford, Oxford, UK; Oxford Centre for Clinical Magnetic Resonance Research, Division of Cardiovascular Medicine, Radcliffe Department of Medicine, University of Oxford, Oxford, UK; School of Biomedical Engineering and Imaging Sciences, King’s College London, 5th Floor Becket House, Lambeth Palace Road, London SE1 7EU, UK; School of Biomedical Engineering and Imaging Sciences, King’s College London, 5th Floor Becket House, Lambeth Palace Road, London SE1 7EU, UK; Department of Cardiovascular Imaging, School of Biomedical Engineering and Imaging Sciences, Guy’s and St Thomas’ NHS Foundation Trust, London, UK; NIHR Oxford Biomedical Research Centre, Division of Cardiovascular Medicine, Radcliffe Department of Medicine, University of Oxford, and Oxford University Hospitals NHS Foundation Trust, Oxford, UK; NIHR Oxford Biomedical Research Centre, Division of Cardiovascular Medicine, Radcliffe Department of Medicine, University of Oxford, and Oxford University Hospitals NHS Foundation Trust, Oxford, UK; Division of Cardiovascular Medicine, University of Virginia Health System, Charlottesville, VA, USA; Department of Cardiovascular Medicine, Heart and Vascular Institute, Cleveland, OH, USA; MedStar Health Research Institute, Georgetown University, Washington, DC, USA; Oxford Centre for Clinical Magnetic Resonance Research, Division of Cardiovascular Medicine, Radcliffe Department of Medicine, University of Oxford, Oxford, UK; NIHR Oxford Biomedical Research Centre, Division of Cardiovascular Medicine, Radcliffe Department of Medicine, University of Oxford, and Oxford University Hospitals NHS Foundation Trust, Oxford, UK; NIHR Oxford Biomedical Research Centre, Division of Cardiovascular Medicine, Radcliffe Department of Medicine, University of Oxford, and Oxford University Hospitals NHS Foundation Trust, Oxford, UK; School of Biomedical Engineering and Imaging Sciences, King’s College London, 5th Floor Becket House, Lambeth Palace Road, London SE1 7EU, UK

**Keywords:** cardiac atlas, clinical biomarker, computational anatomy, machine learning, shape analysis, ventricular remodelling

## Abstract

**Aims:**

Obstructive hypertrophic cardiomyopathy (oHCM) is characterized by dynamic obstruction of the left ventricular (LV) outflow tract (LVOT). Although this may be mediated by interplay between the hypertrophied septal wall, systolic anterior motion of the mitral valve, and papillary muscle abnormalities, the mechanistic role of LV shape is still not fully understood. This study sought to identify the LV end-diastolic morphology underpinning oHCM.

**Methods and results:**

Cardiovascular magnetic resonance images from 2398 HCM individuals were obtained as part of the NHLBI HCM Registry. Three-dimensional LV models were constructed and used, together with a principal component analysis, to build a statistical shape model capturing shape variations. A set of linear discriminant axes were built to define and quantify (*Z*-scores) the characteristic LV morphology associated with LVOT obstruction (LVOTO) under different physiological conditions and the relationship between LV phenotype and genotype. The LV remodelling pattern in oHCM consisted not only of basal septal hypertrophy but a combination with LV lengthening, apical dilatation, and LVOT inward remodelling. Salient differences were observed between obstructive cases at rest and stress. Genotype negative cases showed a tendency towards more obstructive phenotypes both at rest and stress.

**Conclusions:**

LV anatomy underpinning oHCM consists of basal septal hypertrophy, apical dilatation, LV lengthening, and LVOT inward remodelling. Differences between oHCM cases at rest and stress, as well as the relationship between LV phenotype and genotype, suggest different mechanisms for LVOTO. Proposed *Z*-scores render an opportunity of redefining management strategies based on the relationship between LV anatomy and LVOTO.

## Structured Graphical Abstract

**Graphical Abstract:**
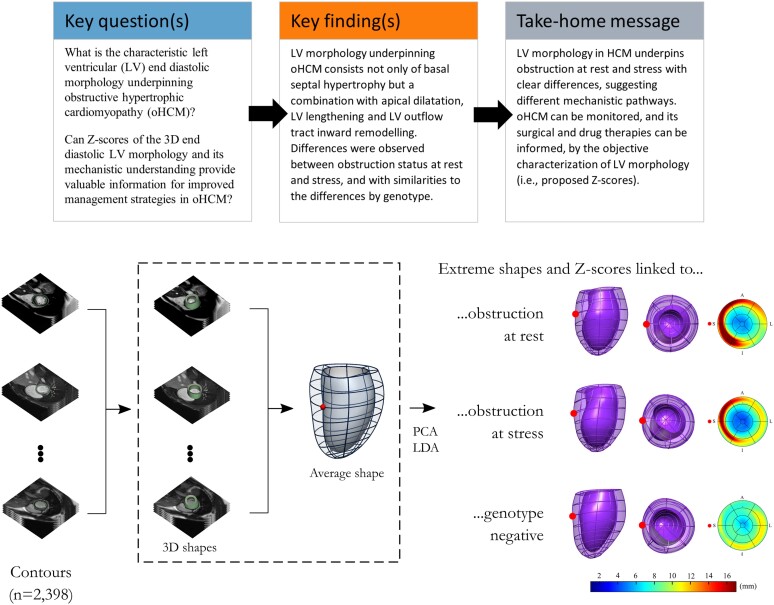

**Translational perspective**

A detailed 3D anatomical assessment is available in the form of a combination of *Z*-scores along each of the linear axes of left ventricular remodelling. Proposed *Z*-scores provide quantitative information on the development and severity of the left ventricular anatomical substrate underpinning left ventricular outflow tract obstruction. There is thus an opportunity for translating findings into better risk management and patient follow up, into improved patient selection, planning, and outcome assessment in surgical septal interventions, or into an objective measure of disease activity to evaluate the efficacy of emerging hypertrophic cardiomyopathy drug therapies.

## Introduction

Obstructive hypertrophic cardiomyopathy (oHCM) is characterized by dynamic obstruction of the left ventricular (LV) outflow tract (LVOT) at rest or under physiological provocation. oHCM is present in ∼20–25% of HCM cases.^1,2^ Known to be a major determinant of adverse clinical outcomes,^3^ LVOT obstruction (LVOTO) is a highly dynamic process with beat-to-beat variability and multifactorial causes. It is considered to be caused by the interplay between the systolic anterior motion of the mitral valve, the hypertrophied septal wall, hypercontractile myocardium, and various papillary muscle abnormalities, such as thickening, bifid character, and anterior displacement.^4–7^ Furthermore, structural mitral valve alterations, such as elongated leaflets and abnormal chordal attachment, are common in HCM, and thought to contribute to adverse outcomes.^8^

Severity assessment in HCM is routinely performed using 2D and Doppler echocardiography. However, echocardiographic assessment commonly results in both under or overestimation of septal wall thickness compared with cardiovascular magnetic resonance (CMR).^9–11^ Moreover, Doppler assessment of LVOTO, aiming to capture peak velocity, suffers from the dynamic nature of LVOTO itself, from limitations in achieving the right probe orientation, and from simplifications of Bernoulli’s formulation.^12^ Therefore, characterizing the anatomical substrate that causes LVOTO by detailed 3D imaging modalities could offer an improved understanding of pathophysiology and complementary diagnostic and therapy monitoring strategies.

Treatment of obstruction in HCM focuses on improving symptoms by pharmacological or mechanical intervention (septal myectomy or alcohol septal ablation).^13^ Recently, mavacamten, a specific cardiac myosin ATPase inhibitor (in Phase 3 trials), has shown promising results, significantly improving LVOTO, exercise capacity, physical function, and quality of life in oHCM patients.^14^ Though not all patients with LVOTO, defined by Doppler measurement of LVOT pressure drop, saw a clinical benefit, highlighting the need to provide further insight into structural and functional mechanisms underpinning outflow obstruction.

Computational cardiac models have emerged as valuable tools to clarify pathophysiological mechanisms and improve treatment planning, as well as diagnosis.^15^ Among those, statistical shape models have been used to study the relationships between LV shape and risk factors^16,17^ or to characterize cardiac remodelling.^18–20^ In HCM, statistical shape models have shown significant differences in end-systolic radial thickness and basal anteroseptal wall geometry between HCM and hypertensive patients^21^ and led to proposal of a non-invasive procedure for genotype positive-phenotype negative prediction.^22^

In this context, this work analyses the different LV phenotypes in oHCM using a detailed statistical shape model. The objective is to improve the mechanistic understanding of the role of LV shape in LVOTO pathophysiology to explore its potential use for improved management strategies.

## Methods

The LV anatomy in HCM, encoded by a statistical shape model, is used to infer the presence of LVOTO, both at rest and stress conditions. The 3D anatomical patterns found are (i) verified in an external cohort, (ii) compared with the patterns of genotype positive vs. negative in HCM, and (iii) interpreted to extract novel mechanistic insights.

### Cohort and data acquisition

The Hypertrophic Cardiomyopathy Registry (HCMR) cohort is a prospective National Heart, Lung, and Blood Institute funded registry of 2750 HCM patients recruited across Europe and North America.^22^ HCMR integrates CMR imaging, biomarkers, and genetic information with standard clinical and echocardiographic data.^23^ CMR data from the HCMR cohort (*n* = 2398) were used to construct a statistical model of the LV.

The HCMR study design and acquisition protocol have been described previously.^22^ Briefly, CMR images were performed at 1.5 or 3 Tesla on MR systems from the three primary vendors (General Electric, Philips Medical Systems, and Siemens Healthcare) using multi-channel phased-array chest coils and electrocardiographic gating. Short-axis cine images were acquired at rest using steady-state free precession imaging, covering the heart in 8 mm thick slices. Cine steady-state free precession imaging parameters included TR/TE 3.1/1.2 ms, in-plane resolution of 2–2.5 mm. Echocardiographic data from routine clinical care within 12 months of the CMR were recorded. This included LVOT pressure drop, estimated using the simplified Bernoulli equation^24^ from continuous-wave Doppler. All HCMR participants provided written informed consent (South Central—Oxford A Research Ethics Committee approval: 14/SC/0190; clinicaltrials.gov identifier: NCT01915615).

HCMR population was divided into four groups to explore differences in their phenotypes: R−: HCM cases at rest (peak pressure drop <30 mmHg at rest); R+: oHCM cases at rest (peak pressure drop ≥30 mmHg at rest); R−S−: HCM cases at rest and stress (peak pressure drop <30 mmHg both at rest and stress); R−S+: oHCM cases at stress but not at rest (peak pressure drop ≥30 mmHg at stress but not at rest). Groups R− and R−S− were analysed separately as not all cases with peak pressure drop measured at rest had a stress test. Cases were also classified according to their genetic information into two extra groups: genotype negative (G−) and genotype positive (G+). G+ cases included mutations of the following genes: sarcomeric genes (MYH7, MYBPC3, TNNT2, TNNI3, MYL2, MYL3, ACTC1, and TPM1) and ‘phenocopy’ genes (GLA, PRKAG2, LAMP2, and TTR).

### Statistical shape analysis

**Figure 1:**
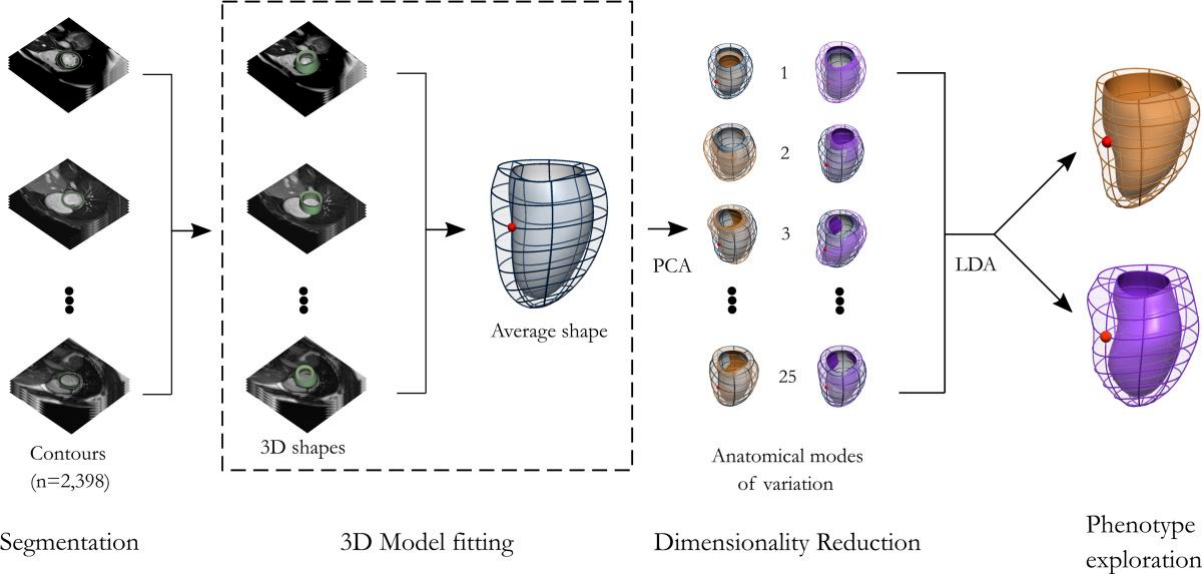
HCMR statistical shape analysis pipeline. From a set of segmented endocardial and epicardial contours, automatic personalized 3D meshes are generated for each patient. (*A*)Statistical shape model is then built using a principal component analysis (PCA), which results in a set of anatomical modes or PCA modes (linear directions of anatomical change from the average shape) that capture the most common shape changes in the cohort. A Fisher linear discriminant analysis (LDA) is finally used to explore the characteristic phenotypes associated with left ventricular outflow tract obstruction in HCM under different conditions.

A statistical shape model of LV anatomy was created using the personalization framework^25,26^ implemented in Matlab (Mathworks, Natick, MA, USA). Three main steps are required to construct a statistical shape model from CMR images: segmentation, 3D model fitting, and dimensionality reduction—see methodological overview in *Figure 1*.

Endocardial and epicardial contours were delineated at end-diastole for each slice of the LV short-axis cine stack acquired at rest using Q-mass (QMassMR, version 8.1; Medis, Leiden, The Netherlands). These contours were then converted into a binary segmentation. A 3D model of each patient was then automatically fitted to the binary mask by warping a template mesh into it.^25,26^ The distance from mesh surface to contours was used to assess mesh fitting accuracy.

Finally, a principal component analysis (PCA) was used to build the statistical shape model from all available HCMR cases with CMR data acquired at rest. PCA finds the directions that maximize the variance in the observed shapes around their average, i.e. the linear directions of anatomical change from the population average, that are called anatomical modes or PCA modes. PCA modes are ranked by the amount of variance explained, so that the first modes represent the most common shape changes in a cohort. As a result of PCA, each anatomy can be described by a set of shape coefficients that define the linear combination of the PCA modes. LV wall thickness was computed for each shape using the personalized 3D models and is shown with a standard American Heart Association’s (AHA) 17-Segment Bull’s eye plot.

### LV phenotype discriminant of obstruction: finding the LVOTO signature

Information of shape is decomposed by the statistical shape model into several PCA modes, and the task is to find their linear combination that best discriminates between different groups (obstructive/non-obstructive at rest and/or stress). Following the methodology described by Varela *et al*.,^27^ a Fisher linear discriminant analysis (LDA) was used to find the optimal anatomical discriminant mode (i.e. LDA mode). Two LDA models were built. The LDA_rest_ model compared LV shape phenotypes between Groups R− (non-obstructive HCM cases at rest) and R+ (obstructive HCM cases at rest). The LDA_Δstress_ aimed to identify the extra cases that show obstruction at stress but not at rest, and was built to discriminate Groups R−S− (non-obstructive HCM cases at rest and stress) and R−S+ (obstructive HCM cases at stress but not at rest). LV anatomies for each subject were then characterized by a single coefficient along each axis (i.e. *Z*-score). Moreover, the interpretation of the extreme phenotypes from each axis allows the extraction of novel mechanistic insights. All PCA modes adding up to a minimum of 90% cumulative explained variance were included in each LDA as relevant information might be captured by smaller modes.

### Robustness of the LVOTO signature

We first evaluate the robustness of the characteristic phenotypes to the choice of threshold for LVOTO. Shape phenotypes of severe obstructive/non-obstructive cases (peak pressure drop ≥50 mmHg) were compared with those of the LDA_rest_ previously found with LVOT peak pressure drop threshold at 30 mmHg.

Then, the LDA_rest_ was externally evaluated with a population of 101 HCM cases from the Oxford Centre for Magnetic Resonance Imaging (OCMR), including 87 non-obstructive HCM cases at rest (86%) and 14 oHCM cases at rest (14%). External evaluation cases were not a subset of the HCMR cohort. All OCMR participants provided written informed consent [REC approval number—NREC (12/LO/1979)]. For more details about the external evaluation population characteristics, the reader is referred to the Supplementary materials.

Finally, to avoid patient selection bias when comparing the findings between the LDA_rest_ and the LDA_Δstress_, an extra LDA_rest_ model was built using only paired cases from the LDA_Δstress_ (i.e. including only cases that have both rest and stress pressure drop measurements).

### LV phenotype–genotype relationship

The relationship between the 3D LV anatomical signatures (i.e. phenotypes) and genotype expression was explored by projecting all HCMR cases with genotype information into the LDA_rest_ and LDA_Δstress_ axes, obtaining associated *Z*-scores.

In addition, an LDA model was built to find the morphological signature that best differentiates genotype negative (G−) vs. genotype positive (G+), the LDA_gen_.

### Statistical analysis

Normality of baseline and imaging characteristics was ensured using a Shapiro–Wilk test and variables are presented as mean (±standard deviation) or as medians (with 25% and 75% percentiles) accordingly. Comparison of continuous variables between two groups was performed using a Mann–Whitney *U* test or unpaired Student’s *t*-test as appropriate. Categorical variables were compared using the Pearson χ^2^ test. Modes of variation are Gaussian distributed by definition. Therefore, a two-tailed Student’s *t*-test was used to determine differences between shape coefficient distributions (i.e. Z-scores) for each group along each axis (both PCA and LDA). *P*-value <0.05 was considered statistically significant. All analyses were performed in Matlab (Mathworks, Natick, MA, USA). Discriminatory performance for each axis was assessed using the area under the receiver operating characteristic (AUC). Potential overfitting was assessed by computing the AUC in resubstitution and leave-one-out cross-validation scenarios.^28^

## Results

### Study population characteristics

**Figure 2:**
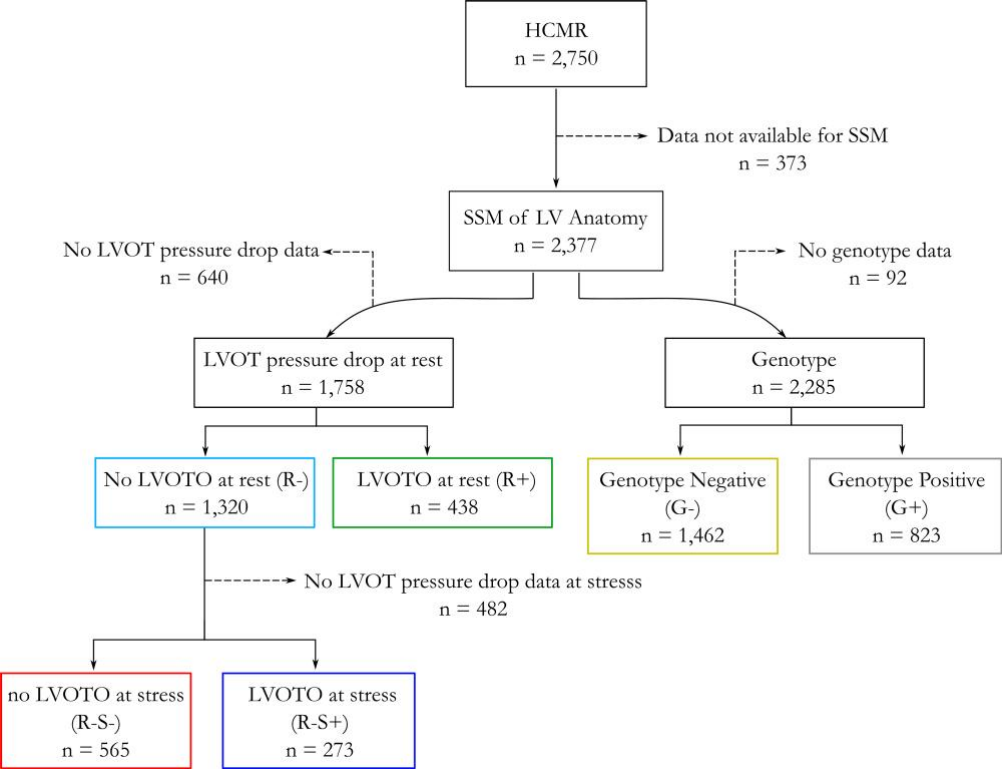
Flow chart of study design resulting in the six different groups studied. HCMR, Hypertrophic Cardiomyopathy Registry; LV, left ventricular; LVOT, left ventricular outflow tract; SSM, statistical shape model.

Of the 2750 cases included in the HCMR cohort, 2377 cases had adequate data for shape modelling. Among those, 1758 cases with LVOT pressure drop measurement at rest were available for the discriminant analysis between Groups R− and R+ (i.e. LDA_rest_), resulting in 1320 non-obstructive (75%, R−) and 438 obstructive cases (25%, R+). Around 838 cases with LVOT pressure drop measurement both at rest and stress were available for the discriminant analysis between Groups R−S− and R−S+ (i.e. LDA_Δstress_), resulting in 565 non-obstructive cases at rest and stress (67%, R−S−) and 273 obstructive cases at stress but not at rest (33%, R−S+). Genotype mutation information was available for 2286 cases, resulting in 1462 genotype negative (64%, G−) and 823 genotype positive (36%, G+). For a visual description, see *Figure 2*.

Baseline and imaging characteristics are described in *Tables 1* and *2*. Obstructive patients at rest (R+) were significantly older, more predominantly male, and had a higher BMI compared with non-obstructive cases at rest (R−). Moreover, R+ cases had a significantly decreased diastolic blood pressure, as well as significantly higher New York Heart Association (NYHA) class. Higher frequency of hypertensive patients was found within the R+ group. Differences in medication were noted between R+ and R− groups except for anticoagulants. In terms of imaging characteristics, obstructive cases at rest (R+) had significantly increased left ventricular end-diastolic volume indexed, left ventricular stroke volume indexed, and left ventricular mass index (LVMI). As expected, R+ cases had increased LVOT pressure drop at rest. In the comparative analysis between R−S− and R−S+ groups, there were significant differences in age and BMI, but not gender. R−S+ cases were associated with increased systolic blood pressure, hypertension, and higher NYHA class. Treatment with beta-blockers and disopyramide was more frequent in the R−S+ group. Finally, a significant increase in LVMI was observed. For a detailed description of the population, the reader is referred to the Supplementary materials.

**Table 1:** HCMR study population baseline characteristics

	Total (*n* = 2377)	HCM_R−_ (*n* = 1320)	oHCM_R+_ (*n* = 438)	HCM_R-S−_ (*n* = 565)	oHCM_R-S+_ (*n* = 273)
Age, years	52 (43–58)	51 (42–58)	53 (46–60)*	49 (41–57)	51 (44–58)**
Male	1693 (71)	981 (74)	262 (60)*	422 (75)	209 (76)
Weight, kg	86.0 (75.0–99.2)	86.5 (75.0–98.7)	89.3 (76.0–102.9)*	84.9 (74.0–96.4)	92.9 ± 17.1**
BMI, kg/m^2^	28.2 (25.2–32.2)	28.4 (25.1–31.9)	30.1 (25.7–33.7)*	27.8 (24.8–31.2)	30.4 (26.6–33.8)**
HR, b.p.m.	64.0 (56.0–72.0)	63.9 (56.0–72.0)	65.1 (57.0–74.0)	64.0 (57.0–72.0)	65.9 (57.8–74.0)
Systolic BP, mmHg	129.0 (92.9–218.0)	129.7 (117.0–140.0)	127.4 ± 21.7	127.6 (115.0–138.0)	133.3 ± 29.1**
Diastolic BP, mmHg	77.0 (55.4–104.7)	77.8 (69.0–85.0)	74.2 (67.0–82.0)*	77.6 (69.0–85.0)	77.6 ± 14.6
Sarcomere mutation positive	823 (36)	474 (37)	115 (28)*	221 (41)	68 (26)*
NYHA class					
ȃI	1562 (67)	871 (67)	204 (48)*	384 (69)	135 (50)**
ȃII	618 (26)	360 (28)	155 (36)*	149 (27)	108 (40)**
ȃIII/IV	164 (7)	68 (5)	69 (16)*	21 (4)	22 (8)**
Family history SCD					
ȃ1st degree	295 (12)	172 (13)	43 (10)	78 (14)	29 (11)
ȃ2nd degree	289 (12)	167 (13)	39 (9)*	74 (13)	32 (12)
Syncope	322 (14)	185 (14)	67 (15)	80 (14)	37 (14)
NSVT	172 (12)	116 (13)	27 (11)	55 (15)	22 (13)
Smoker	306 (13)	177 (13)	57 (13)	78 (14)	32 (12)
AF	270 (11)	143 (11)	58 (13)	55 (10)	23 (8)
Hypertension	858 (36)	459 (35)	196 (45)*	171 (30)	125 (46)**
Diabetes	195 (8)	101 (8)	36 (8)	31 (6)	22 (8)
Medications					
ȃBeta-blockers	1331 (56)	720 (55)	327 (75)*	287 (51)	192 (71)**
ȃCCB	450 (17)	246 (19)	102 (23)*	92 (16)	57 (21)
Disopyramide	69 (3)	26 (2)	35 (8)*	4 (1)	11 (4)**
Anticoagulant	215 (9)	110 (8)	39 (9)	49 (9)	13 (5)
ACE/ARB	572 (22)	313 (24)	83 (19)*	125 (22)	62 (23)
Diuretics	273 (10)	138 (11)	66 (15)*	51 (9)	30 (11)
Amiodarone	41 (2)	14 (1)	14 (3)*	6 (1)	2 (1)
Statin	369 (16)	198 (15)	91 (21)*	82 (15)	59 (22)

Values are given as mean ± SD or median (25–75% percentiles) or *n* (% with respect to number of valid cases).

ACE/ARB, angiotensin-converting enzyme/angiotensin receptor blocker; AF, atrial fibrillation; BMI, body mass index; BP, blood pressure; CCB, calcium channel blocker; HCM, hypertrophic cardiomyopathy; HCM_R-_, HCM non-obstructive cases at rest; HR, heart rate; NSVT, non-sustained ventricular tachycardia; NYHA, New York Heart Association; oHCM, obstructive hypertrophic cardiomyopathy; oHCM_R+_, HCM obstructive cases at rest; HCM_R-S−_, HCM non-obstructive cases at rest and stress; oHCM_R-S+_, HCM obstructive cases at stress but not at rest; SCD, sudden cardiac death.

*P* < 0.05 with respect to Group R−.

*P* < 0.05 with respect to Group R−S−. For more details, including the number of valid cases for each variable, see Supplementary materials. From the total number of cases in the HCMR cohort, only those used for discriminant analysis are reported.

**Table 2:** Study population imaging characteristics

	Total (*n* = 2377)	HCM_R−_ (*n* = 1320)	oHCM_R+_ (*n* = 438)	HCM_R-S−_ (*n* = 565)	oHCM_R-S+_ (*n* = 273)
Mean LVWT, mm	8.5 (7.4–10.0)	8.7 (7.4–10.0)	8.8 (7.5–9.9)	8.7 (7.3–10.1)	8.9 (7.5–10.3)
Max LVWT, mm	16.9 (14.2–20.3)	17.5 (14.4–20.7)	17.5 (14.6–20.1)	17.4 (14.2–20.8)	17.8 (14.7–20.7)
LVEDV, mL	169.1 (143.8–195.3)	167.3 (141.6–196.9)	175.1 ± 42.5*	169.3 ± 39.9	176.5 ± 42.5**
LVEDVI, mL/m^2^	84.2 (73.8–94.2)	83.3 (73.1–93.7)	86.4 (75.8–96.3)*	83.3 (48.9–93.4)	86.9 ± 16.7
LVESV, mL	60.9 (44.7–75.9)	59.7 (44.6–74.1)	62.9 (44.8–78.5)	59.3 (44.5–74.1)	59.9 (45.0–74.1)
LVESVI, mL/m^2^	30.1 (22.9–36.7)	29.8 (22.7–36.2)	30.9 (23.4–37.7)	29.7 (23.0–36.0)	28.9 (21.9–35.8)
LVSV, mL	107.2 (94.3–121.9)	106.3 (93.3–121.2)	110.3 (96.0–125.0)*	105.3 (93.4–119.0)	111.9 ± 25.3**
LVSVI, mL/m^2^	53.7 (47.5–60.1)	53.2 (47.2–59.9)	55.0 (48.2–61.6)*	53.2 (47.3–59.8)	53.7 (47.1–60.9)
LVEF, %	64.6 (59.2–70.2)	64.7 (59.5–70.0)	64.5 (58.8–70.5)	64.6 (59.3–69.8)	65.7 (60.7–71.4)
LVM, g	165.1 (128.8–199.3)	159.0 (125.6–190.4)	171.7 ± 58.6*	156.9 (125.2–183.7)	198.0 ± 70.2**
LVMI, g/m^2^	81.6 (65.5–96.7)	78.9 (64.3–92.4)	94.4 (74.3–115.3)*	78.3 (63.9–91.3)	97.1 ± 30.0**
LGE	1169 (50)	627 (48)	208 (48)	673 (52)	190 (44)
LVOT drop rest, mmHg		7.9 (3.0–12.0)	64.1 (42.1–82.8)*	5.6 (0.0–8.0)	15.3 (9.9–21.0)**
LVOT drop stress, mmHg				8.6 (2.0–14.0)	64.2 (42.0–85.0)**

Values are given as mean ± SD or median (25–75% percentiles) or *n* (% with respect to number of valid cases).

HCM, hypertrophic cardiomyopathy; HCM_R-_, HCM non-obstructive cases at rest; HCM_R-S−_, HCM non-obstructive cases at rest and stress; LVWT, left ventricular wall thickness; LVEDV, left ventricular end-diastolic volume; LVEDVI, left ventricular end-diastolic volume indexed; LVESV, left ventricular end-systolic volume; LVESVI, left ventricular end-systolic volume indexed; LVSV, left ventricular stroke volume; LVSVI, left ventricular stroke volume indexed; LVEF, left ventricular ejection fraction; LVM, left ventricular mass; LVMI, left ventricular mass index; LGE, presence of late gadolinium enhancement; LVOT, left ventricular outflow tract; oHCM, obstructive hypertrophic cardiomyopathy; oHCM_R+,_ HCM obstructive cases at rest; oHCM_R-S+_, HCM obstructive cases at stress but not at rest. Mean and Max LVWT measures derived from the personalized 3D meshes. The rest of imaging characteristics were derived from CMR images.

*P* < 0.05 with respect to Group R−.

*P* < 0.05 with respect to Group R−S−. For more details, including the number of valid cases for each variable, see Supplementary materials. From the total number of cases in the HCMR cohort, only those used for discriminant analysis are reported.

### The statistical shape model

In the construction of the statistical shape model, meshes were fitted with an error distance close to half the CMR images resolution (average fitting error of 1.0 ± 0.4 mm). PCA resulted in the first 25 modes explaining 91% of the shape variability in the population.

### The LV anatomical features associated with obstruction at rest

**Figure 3:**
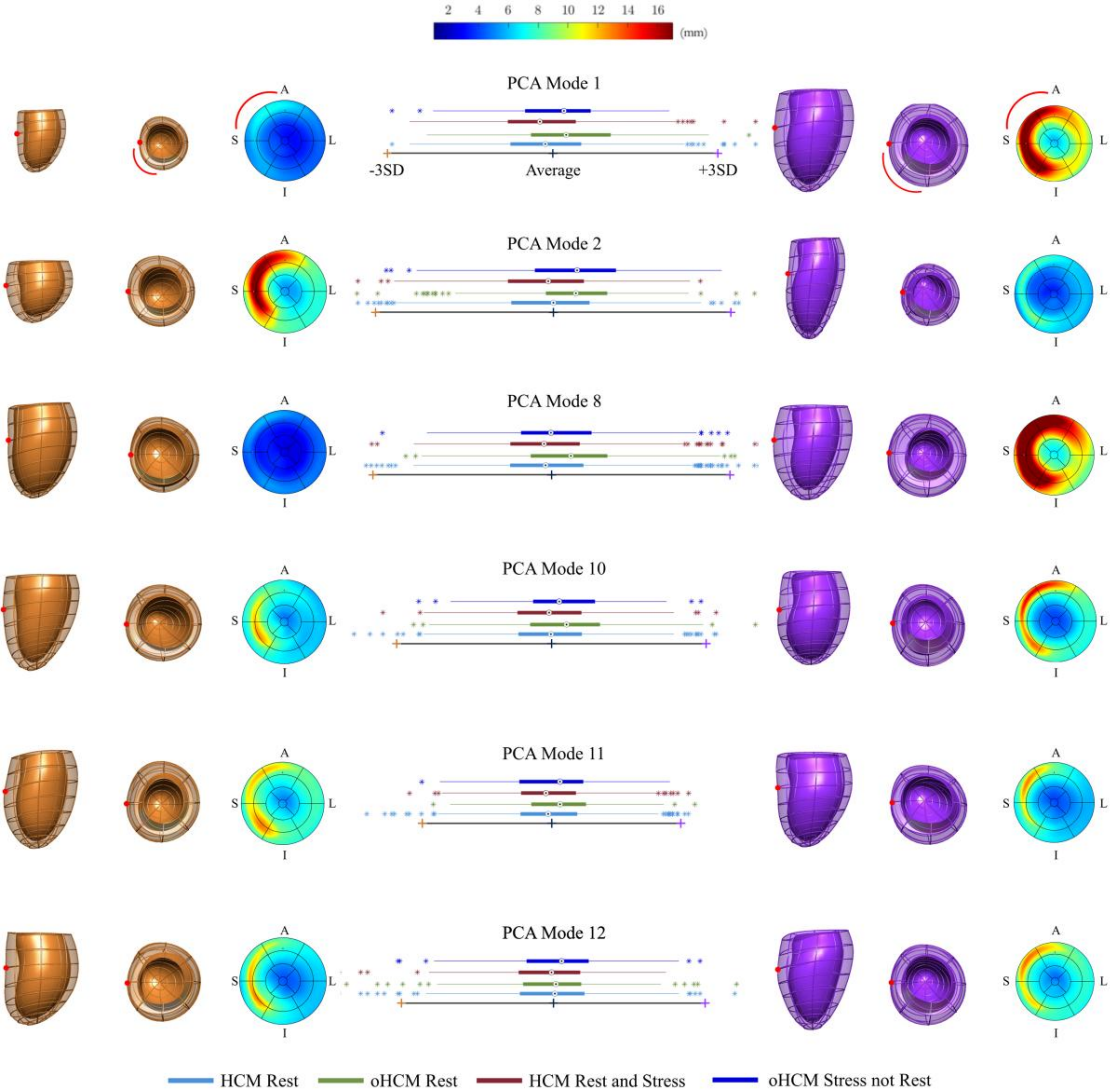
HCMR statistical shape analysis. Most relevant PCA modes of variation showing significant differences between groups. Box-plots represent the distribution of Z-scores along each axis for each group: 1. Non-obstructive cases at rest (blue/first row – R−); 2. Obstructive cases at rest (green/second row – R+); 3. Non-obstructive cases at rest and stress (red/third row – R−S−); 4. Obstructive cases at stress but not at rest (dark blue/fourth row – R−S+). The average of the population is represented by a dark blue/middle cross (Z-score 0). Orange/left cross and shapes show −3 SD from the average shape; purple/right cross and shapes, +3 SD. Mode 1 shows changes in LV size and length, as well as basal septal hypertrophy. Mode 2 captured LV length and sphericity variations. Mode 8 captured a global concentric remodelling, with marked septal hypertrophy. Mode 10 shows the presence of apical dilatation. Mode 11 shows curvature changes around the septum. Mode 12 captured changes in the septal wall thickening location. LVOT location is clarified with a red arch (note Bull's eye plot (right) is viewed from the apex, model views are from lateral (left) and base (middle)). Red dot: septal wall location. A, Anterior; I, Inferior; L, Lateral; S, Septal. Maximum wall thickness in the AHA 17-Segment Bull's eye plot is 17 mm.

PCA Modes 1, 2, 4, 8, 10, 11, 15, 16, and 18 differed significantly (*P* < 0.05) between non-obstructive HCM cases at rest (i.e. Group R−) and oHCM cases at rest (i.e. Group R+). PCA Mode 1 captured changes in LV size and length, together with marked basal septal hypertrophy. LV lengthening without septal hypertrophy was captured by Mode 2. LV sphericity was also captured by Mode 2. Mode 8 shows a global concentric remodelling, with marked septal hypertrophy. Mode 10 captured the presence of apical dilatation with a small degree of focal hypertrophy near the LVOT. Finally, Mode 11 captured septal curvature changes. See *Figure 3* for a detailed visual representation of the relevant anatomical modes.

**Figure 4:**
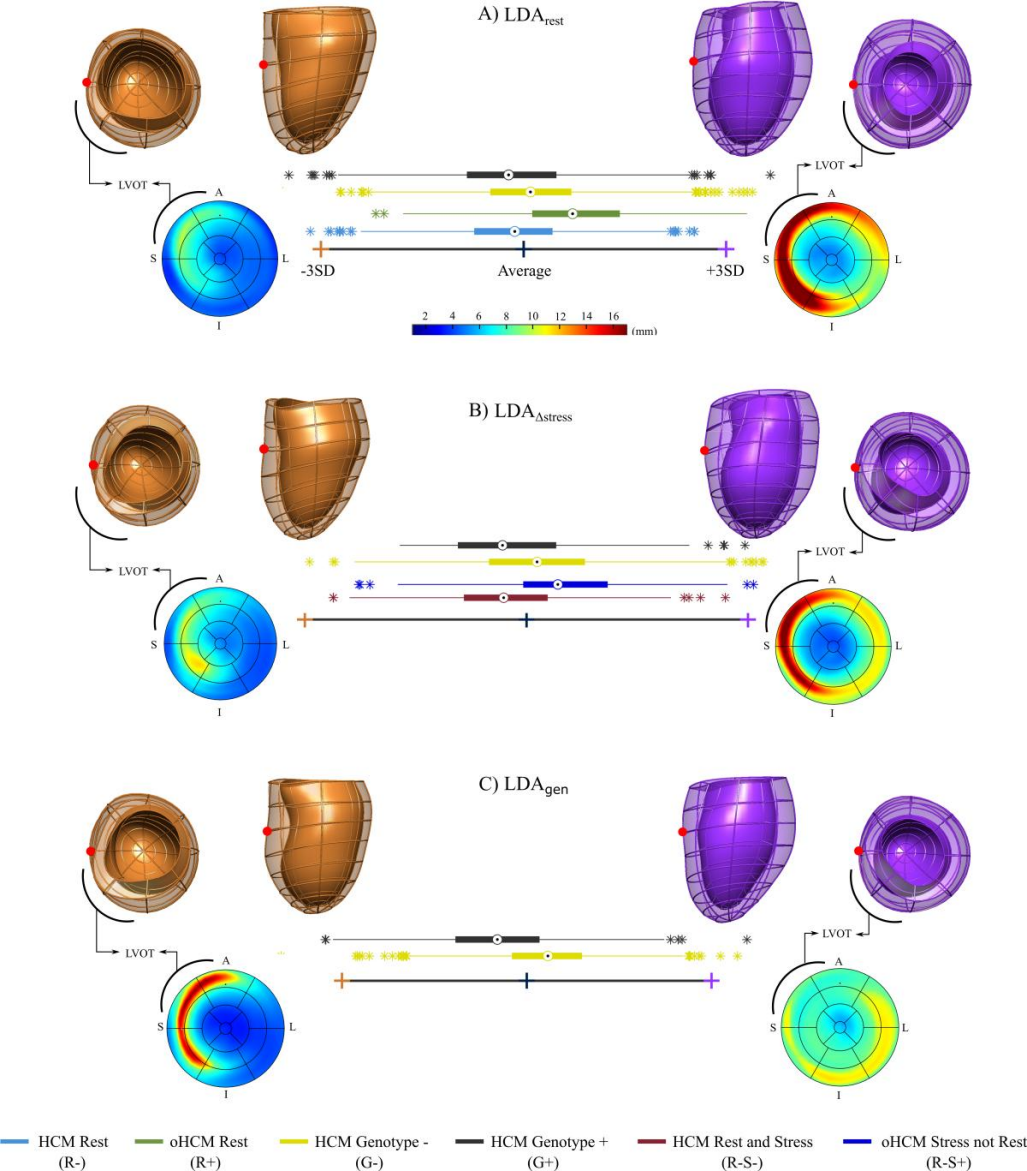
HCMR signature of LVOTO. Set of linear discriminant axes that characterize the role of LV shape in obstructive HCM (oHCM). Boxplots represent the Z-score distributions along each axis for each group. Orange/left cross and 3D shapes show −3SD from the average shape (blue/middle cross); purple/right cross and shape, +3SD. (*A*) LDA_rest_: Linear discriminant axis between non-obstructive HCM cases at rest (blue/first row – R−) and obstructive HCM (oHCM) cases at rest (green/second row – R+). (*B*) LDA_∆stress_: Linear discriminant axis between non-obstructive HCM cases at rest and stress (red/first row – R−S−) and obstructive HCM cases at stress but not at rest (dark blue/second row – R−S+). Genotype negative (yellow/third row – G−) and genotype positive (black/fourth row – G+) scores are projected along the LDA_rest_ and LDA_∆stress_. (*C*) LDA_gen_: Linear discriminant axis between genotype negative (yellow/first row – G−) and genotype positive (black/second row – G+) cases. Black arch: LVOT location (note Bull's eye plot (bottom) is viewed from the apex, model views are from lateral (top-left) and base (top-right)). Red dot: Septal wall location. A, Anterior; I, Inferior; L, Lateral; S, Septal.

The LDA_rest_ discriminated cases with an AUC of 0.76 in resubstitution and 0.74 in leave-one-out cross-validation. LDA_rest_ extreme shapes (see *Figure 4A*) show increased septal thickness, apical dilatation, and LV lengthening: oHCM cases at rest tend to have a marked thickening of the anteroseptal and inferoseptal wall, and a dilated LV apex, resulting in increased septal wall curvature. Non-obstructive HCM cases at rest showed a more conical LV shape with reduced hypertrophy.

### The LV anatomical features associated with obstruction at stress

In the search for the anatomy associated with obstruction at stress, PCA Modes 1, 2, 10, and 12 showed significant differences between Groups R−S− and R−S+ (*P* < 0.05), capturing changes in the location of the septal wall thickening (from inferoseptal to anteroseptal wall) as well as apical dilatation changes. The LDA_Δstress_ discriminated R−S− and R−S+ cases with an AUC of 0.74 in resubstitution and 0.70 in leave-one-out cross-validation. oHCM cases tended to have a marked spiral thickening pattern that starts at the anteroseptal wall and extends to the inferoseptal wall from base to mid (see *Figure 4B*), and a clear inward remodelling (i.e. narrowing) of the LVOT. As seen in the LDA_rest_, oHCM cases of the LDA_Δstress_ tended to have a dilated LV apex, resulting in increased septal wall curvature. Non-obstructive HCM cases showed a more conical LV shape with a focal degree of hypertrophy at the middle inferoseptal wall, as well as a marked basal outward remodelling (i.e. widening) of the LVOT.

### Robustness of the LV anatomical signatures of LVOTO

The LDA_rest_ model built with a different threshold of obstruction (peak pressure drop ≥50 mmHg instead of ≥30 mmHg) discriminated cases with the same performance (AUC of 0.76 in resubstitution and 0.74 leave-one-out cross-validation) and its extreme 3D phenotypes showed an almost identical shape pattern compared with the 30 mmHg LDA_rest_ (see Supplementary data online, *Figure S1*).

Mesh personalization of the external evaluation cohort was performed with an average fitting error of 1.1 ± 0.3 mm (approximately half of the CMR image resolution). The LDA_rest_ (with 30 mmHg threshold) externally discriminated external cases with an AUC of 0.72, a small drop of performance from the 0.74 AUC cross-validated in the HCMR population.

The extra LDA_rest_ model (with 30 mmHg threshold) built using paired cases from the LDA_Δstress_ showed the same qualitative characteristics as the original LDA_rest_ model, but with milder thickening patterns in the obstructive extreme shape (i.e. +3SD from the average shape; see Supplementary data online, *Figure S2*).

### Relationship between LV phenotype, genotype, and presence of obstruction

G− cases displayed a more obstructive anatomy in relation to both the LDA_rest_ (0.12 ± 0.98 vs. −0.18 ± 1.0, *P* < 0.05) and LDA_Δstress_ (0.16 ± 0.98 vs. −0.26 ± 0.99, *P* < 0.05) *Z*-scores when compared with G+.

The LDA_gen_ discriminated G+ vs. G− cases with an AUC of 0.75 in resubstitution and 0.73 in leave-one-out cross-validation. G+ cases tended to show a marked spiral thickening pattern (see *Figure 4C*), a clear outward remodelling (i.e. widening) of the base towards the LVOT, and a marked apical dilatation. G− cases tended to show a pattern with narrowing of the LVOT (i.e. inward remodelling of the basal anteroseptal wall) and mild thickening of the inferolateral wall.

## Discussion

Using a novel SSM analysis of LVOTO, we found that LV morphology in oHCM at end-diastole is associated with not only the well-recognized basal septal hypertrophy, but also a combination with LV lengthening, apical dilatation, and inward remodelling (i.e. narrowing) of the LVOT. Despite sharing most of these features, there are salient differences between rest and stress in this LV morphology. Genotype negative HCM subjects had higher *Z*-scores than the genotype positive subjects, both at rest and stress.

### LV phenotype heterogeneity in HCM: beyond anteroseptal basal hypertrophy

Anteroseptal basal septal thickening is commonly acknowledged as the main phenotypic LV feature in oHCM.^5,23^ However, our results showed LV shape differences between obstructive and non-obstructive HCM patients were not restricted to thickening changes of the basal anteroseptal wall: cases with LVOTO at rest (R+) tend to have larger LV with hypertrophied basal anteroseptal and inferoseptal wall and apical dilatation compared with non-obstructive cases (R−). This combination leads to a change in septal curvature, with oHCM patients having a marked isolated basal septal morphology and narrower LVOT.

The LV phenotype of oHCM cases with LVOTO only at stress and not at rest (R−S+) shared most of these features. However, an unexpected marked inferoseptal (not anteroseptal where the LVOT is) thickening of the basal and mid wall was captured by the LDA_rest_, whereas the LDA_Δstress_ displayed a spiral pattern of LV hypertrophy, in concordance with previous reports that described the spiral pattern as an independent predictor of LVOTO.^29^ Moreover, the LDA_Δstress_ showed the obstructive extreme with a more pronounced inward remodelling of the basal anteroseptal wall (i.e. LVOT narrowing) compared with the LDA_rest_.

As in previous reports, we observed baseline differences between groups. Genotype negative (G−) HCM is known to be associated with a more obstructive phenotype.^23^ Moreover, G− patients are known to be older, more predominantly males, hypertensive, and with higher BMI. Modifiable risk factors such as diastolic blood pressure are associated with a greater odds of genotype negative (G−) HCM,^30^ which could explain the observed differences in diastolic blood pressure in our cohort. Although diastolic blood pressure was lower in patients with resting obstruction (R+), the higher hypertension frequency likely reflects the effect of treatment. In terms of imaging characteristics, LVMI differences between obstructive and non-obstructive groups both at rest and stress were expected given the higher frequency of G− cases, which are known to have a higher degree of hypertrophy.^23^ Moreover, increased LVMI in LVOT obstruction would be expected as an adaptation to pressure load.

### Robustness of observed LV phenotypes

The generalization of the LDA_rest_ was verified in an external cohort (drop of AUC from 0.74 to 0.72 in the internal cross-validation to the external cohort). Such generalization is grounded on the diversity and sample size of the HCMR cohort, which results in robust LVOTO-related features captured by the LDA_rest_. The external validation of the LDA_Δstress_ was not possible, because only information at rest for the external validation cohort was available.

Results are also robust to the choice of LVOTO threshold (≥30 or ≥50 mmHg, see Supplementary data online, *Figure S1*), specially with respect to the unexpected inferoseptal thickening of the basal and mid wall for LVOTO by the LDA_rest_—this is thus an intriguing finding, given the anteroseptal direction of the LVOT and MV, that may be indicative of a more widespread interaction between geometry and function which warrants further study.

### Relationship between LV phenotype and genotype in HCM

Genotype negative subjects have been reported to be more obstructive than genotype positive subjects.^23^ Our results are consistent, since they show genotype negative subjects with an anatomical substrate associated with LVOTO (i.e. larger *Z*-scores along both the LDA_rest_ and LDA_Δstress_ axes).

The discriminant axis between G+ and G− patients showed clear differences with a mixed combination of anatomical features associated with obstruction: whereas G+ cases showed a localized thickening in the anteroseptal wall and apical dilation, G− cases show a narrowing of the LVOT. These findings suggest that different genotypes are associated with different mechanisms that lead to obstruction, and as such they might require tailored surgical/treatment strategies.

### Mechanisms of LVOTO driven by LV shape

The role of a hypertrophied basal anteroseptal wall and inward remodelling of the basal anteroseptal wall, reducing the LVOT area and increasing drag and Venturi forces (i.e. increasing systolic anterior motion of the MV), is recognized as a key mechanism for LVOTO in HCM.^3–6^ Its presence decreases the distance between the septum and elongated MV leaflets, increasing contact chances between them, as well as Venturi forces, which may help sustain LVOTO.

**Figure 5:**
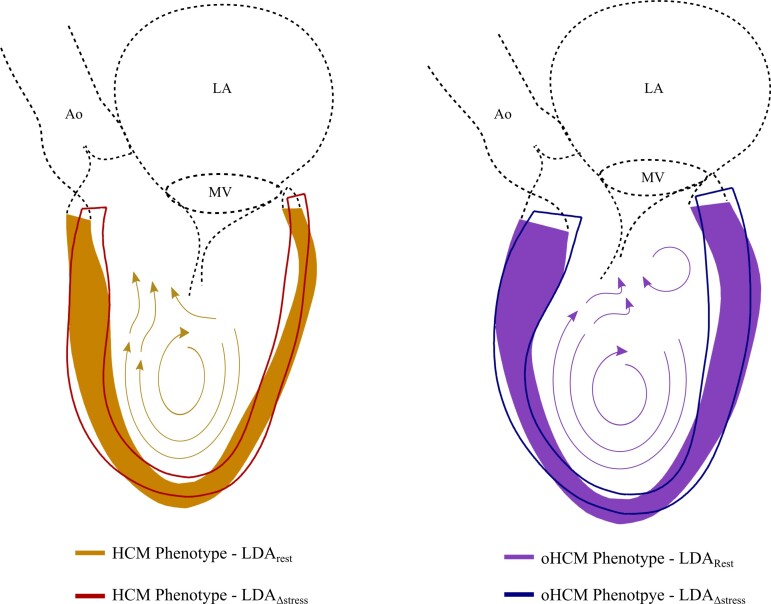
LV shape and LVOTO. Impact of LV shape on vortex formation during early systole, which changes the angle of attack of blood flow with respect to the mitral valve leaflets, increasing the systolic anterior motion of the mitral valve and consequently, LVOTO. Orange/left shape represents −3SD from the average shape along the LDA_rest_ axis (extreme non-obstructive HCM phenotype at rest). Purple/right shape, +3SD (obstructive HCM phenotype at rest). Phenotypes resulting from the LDA_∆stress_ are overlayed with lines. An estimation of the left atrium, mitral valve, aorta, and aortic valve is shown with dotted lines.

However, the observed shape phenotypes were not restricted to such basal wall changes. A further mechanism identified in this study as a potential cause for LVOTO is apical dilatation. Previous studies have shown that LV shape plays a central role in vortex formation within the ventricle and can have a direct impact on the angle of attack of blood flow with respect to the MV leaflets, increasing systolic anterior motion of the MV and consequently LVOTO.^31,32^ Similarly, LV length may also contribute to perturbed blood fluid dynamics and can modify vortex formation (see *Figure 5*). Thus, we provide novel insights into alternate mechanisms underlying LVOTO, not previously been described in the context of HCM.

This mechanistic explanation of the relationship between LV anatomy and LVOTO suggests LV shape could be part of the causal path in oHCM. However, this cannot be proved using the presented SSM. Distal LV cavity dilatation has been shown to progress with longstanding untreated LVOTO^33^ and in cases with mid-cavity obstruction, aneurysmal apical dilatation has been reported.^34^ In such cases, apical dilatation has been shown to improve after palliation of obstruction, for example through pacing, suggesting it may be a consequence, rather than cause, of obstruction.^35^ However, the potential impact of apical dilatation on LV blood flow dynamics, in combination with basal septal hypertrophy and lengthening, suggests that it might not be a consequence but rather part of the causal path in LVOTO. Therefore, we hypothesize that there might be a negative feedback loop between these, and that obstruction begets obstruction in the presence of apical dilation (see *Figure 5*).

### Future work: the role of LV shape in oHCM management

The objective characterization of LV anatomies in oHCM revealed differences in the spectrum of LV phenotypes in cases with obstruction at rest and stress. Given the role of LV shape as the main target for surgical strategies in HCM, the presented methodology could play a role for improved personalized treatment of HCM cases with LVOTO. The presented detailed 3D assessment in the form of a combination of *Z*-scores along each axis of LV remodelling can provide quantitative information on the development and severity of the LV anatomical substrate underpinning LVOTO. Therefore, we have made available for the clinical and scientific community such metrics in the form of a web service (hosted at the Cardiac Atlas Project, www.cardiacatlas.org). Although recent work has shown the potential of simpler metrics derived from three-chamber longitudinal views of the heart for the assessment of LVOTO in HCM,^36^ our 3D analysis provides more information and can be similarly integrated into the clinical workflow in a fully automated manner. There is an opportunity to explore the assessment of the presented 3D LV phenotypes with data already available in current clinical practice. Future studies could explore the relationship between LV shape and LVOTO progression, characterizing the temporal remodelling changes associated with LVOTO. Moreover, 4D flow magnetic resonance imaging could be used to study the relationship between LV shape and intracardiac blood flow, which could provide further insights into some of the presented hypotheses.^37^ Such functional assessment could be then used in combination with detailed anatomical characterization of the LV to improve patient selection and procedural planning for septal myectomy or septal alcohol ablation strategies, procedures known to be highly dependent on surgeon and centre expertise.^38^ Finally, shape analysis, such as the one performed in this study, could be relevant in longitudinal studies of emerging HCM-targeted drugs for patients with obstruction, where cardiac remodelling could be assessed in detail to understand how pharmacological resolution of LVOTO might impact LV shape,^39^ potentially improving long-term outcomes.

### Limitations

Firstly, LVOTO is not only associated with LV shape changes, but is a more complex mechanism. Therefore, not considering any information from the MV, the papillary muscles, regional wall motion abnormalities, or alterations of myocardial mechanics, is a key limitation. Secondly, HCMR echocardiographic data were not routinely collected at the time of CMR. Due to the dynamic nature of LVOTO, changes in the timing of pressure drop assessment might result in variable classification. Similarly, alterations in medical therapy can introduce variability in patient classification based on a single pressure drop measurement. Thirdly, <25% of the HCMR cohort had LVOTO at rest, which could impact the LV shape variability captured by the statistical shape model.

## Conclusion

LV 3D anatomy in obstructive HCM consists not only of the well-recognized basal anteroseptal hypertrophy but a combination with apical dilatation, LV lengthening and inward remodelling of the LVOT. Moreover, differences between LV phenotypes of cases with oHCM at rest and those only at stress were observed, as well as between genotype positive and negative cases. These results suggest potential opportunities of redefining management strategies based on the relationship between LV anatomy and functional obstruction, and the hypothesis that LVOTO begets LVOTO in the presence of apical dilation.

## Supplementary material

Supplementary materials are available at *European Heart Journal – Cardiovascular Imaging* online.

## Supplementary Material

jeac233_Supplementary_Data

## Data Availability

In order to provide a simple tool for LDA_rest_, LDA_Δstress_, and LDA_gen_*Z*-score calculation, meshes and models are available in FigShare (https://figshare.com/s/211f7175f2d0a34b40aa), and a web service (Cardiac Atlas Project, www.cardiacatlas.org) will be released up to publication.
