# Supplementary material for: Left ventricular anatomy in obstructive hypertrophic cardiomyopathy: beyond basal septal hypertrophy
Source: Eur Heart J Cardiovasc Imaging. 2022 Nov 28;24(6):807–18. doi: 10.1093/ehjci/jeac233 (PMC10229266; doi:10.1093/ehjci/jeac233)
Supplement: jeac233_Supplementary_Data [file jeac233_supplementary_data.zip › Supplementary Materials (8).docx]

Supplementary materials

Left Ventricular Anatomy in Obstructive Hypertrophic Cardiomyopathy: Beyond Basal Septal Hypertrophy

Uxio Hermida^a^ MSc, David Stojanovski^a^ MEng, Betty Raman^b^ MD DPhil, Rina Ariga^b^ MBBS DPhil, Alistair A. Young^a^ PhD, Valentina Carapella^a^ PhD, Gerry Carr-White^c^ MD PhD, Elena Lukaschuk^d^ MSc, Stefan K Piechnik^d^ DSc PhD, Christopher M. Kramer^e^ MD , Milind Y. Desai^f^ MD, William S. Weintraub^g^ MD, Stefan Neubauer^b,d^ MD, Hugh Watkins^d^ MD PhD, Pablo Lamata^a^ PhD, on behalf of the HCMR investigators

**Table 1. HCMR baseline characteristics – Groups R- and R+.**

|  | **Total**  **(n = 2,377)** | **Valid** |  | **HCM_R-_**  **(n = 1,320)** | **Valid** |  | **oHCM_R+_**  **(n = 438)** | **Valid** |
| --- | --- | --- | --- | --- | --- | --- | --- | --- |
| Age, yrs | 52 (43-58) | 2,337 |  | 51 (42-58) | 1,292 |  | 53 (46-60)* | 428 |
| Male | 1,693 (71) | 2,376 |  | 981 (74) | 1,320 |  | 262 (60)* | 438 |
| Weight, kg | 86.0 (75.0-99.2) | 2,372 |  | 86.5 (75.0-98.7) | 1,318 |  | 89.3 (76.0-102.9)* | 438 |
| BMI, kg/m2 | 28.2 (25.2-32.2) | 2,373 |  | 28.4 (25.1-31.9) | 1,318 |  | 30.1 (25.7-33.7)* | 437 |
| HR, bmp | 64.0 (56.0-72.0) | 2,220 |  | 63.9 (56.0-72.0) | 1,243 |  | 65.1 (57.0-74.0) | 399 |
| Systolic BP, mmHg | 129.0 (92.9-218.0) | 2,143 |  | 129.7 (117.0-140.0) | 1,188 |  | 127.4±21.7 | 383 |
| Diastolic BP, mmHg | 77.0 (55.4-104.7) | 2,142 |  | 77.8 (69.0-85.0) | 1,187 |  | 74.2 (67.0-82.0)* | 383 |
| Sarcomere mutation positive | 823 (36) | 2,285 |  | 474 (37) | 1,270 |  | 115 (28)* | 414 |
| NYHA class |  | 2,340 |  |  | 1,299 |  |  | 428 |
| I | 1,562 (67) |  |  | 871 (67) |  |  | 204 (48)* |  |
| II | 618 (26) |  |  | 360 (28) |  |  | 155 (36)* |  |
| III/IV | 164 (7) |  |  | 68 (5) |  |  | 69 (16)* |  |
| Family history SCD |  | 2,366 |  |  | 1,312 |  |  | 436 |
| 1st degree | 295 (12) |  |  | 172 (13) |  |  | 43 (10) |  |
| 2nd degree | 289 (12) |  |  | 167 (13) |  |  | 39 (9)* |  |
| Syncope | 322 (14) | 2,369 |  | 185 (14) | 1,314 |  | 67 (15) | 436 |
| NSVT | 172 (12) | 1,436 |  | 116 (13) | 871 |  | 27 (11) | 246 |
| Smoker | 306 (13) | 2,368 |  | 177 (13) | 1,313 |  | 57 (13) | 436 |
| AF | 270 (11) | 2,367 |  | 143 (11) | 1,314 |  | 58 (13) | 436 |
| Hypertension | 858 (36) | 2,369 |  | 459 (35) | 1,314 |  | 196 (45)* | 436 |
| Diabetes | 195 (8) | 2,369 |  | 101 (8) | 1,313 |  | 36 (8) | 436 |
| Medications |  |  |  |  |  |  |  |  |
| β-blockers | 1331 (56) | 2,362 |  | 720 (55) | 1,310 |  | 327 (75)* | 435 |
| CCB | 450 (17) | 2,362 |  | 246 (19) | 1,310 |  | 102 (23)* | 435 |
| Disopyramide | 69 (3) | 2,362 |  | 26 (2) | 1,310 |  | 35 (8)* | 435 |
| Anticoagulant | 215 (9) | 2,362 |  | 110 (8) | 1,310 |  | 39 (9) | 435 |
| ACE/ARB | 572 (22) | 2,362 |  | 313 (24) | 1,310 |  | 83 (19)* | 435 |
| Diuretics | 273 (10) | 2,362 |  | 138 (11) | 1,310 |  | 66 (15)* | 435 |
| Amiodarone | 41 (2) | 2,362 |  | 14 (1) | 1,310 |  | 14 (3)* | 435 |
| Statin | 369 (16) | 2,362 |  | 198 (15) | 1,310 |  | 91 (21)* | 435 |

Values are given as mean±SD or median (25% - 75% percentiles) or n (% with respect to number of valid cases). From the total number of cases in the HCMR cohort, only those used for discriminant analysis are reported. *P-value<0.05 compared to R- group. HCM: Hypertrophic Cardiomyopathy; oHCM: Hypertrophic obstructive cardiomyopathy. BMI: Body mass index; HR: Heart rate; BP: Blood pressure; NYHA: New York Heart Association; SCD: Sudden cardiac death; NSVT: Non-sustained ventricular tachycardia; AF: Atrial fibrillation; CCB: Calcium channel blocker; ACE/ARB: Angiotensin-converting enzyme/angiotensin receptor blocker. HCMR group R- (HCM_R-_): HCM non-obstructive cases at rest; HCMR group R+ (oHCM_R+_): HCM obstructive cases at rest.

**Table 2. HCMR baseline characteristics – Groups R-S- and R-S+.**

|  | **Total**  **(n = 2,377)** | **Valid** |  | **HCM_R-S-_**  **(n = 565)** | **Valid** |  | **oHCM_R-S+_**  **(n = 273)** | **Valid** |
| --- | --- | --- | --- | --- | --- | --- | --- | --- |
| Age, yrs | 52 (43-58) | 2,337 |  | 49 (41-57) | 550 |  | 51 (44-58)** | 270 |
| Male | 1,693 (71) | 2,376 |  | 422 (75) | 565 |  | 209 (76) | 273 |
| Weight, kg | 86.0 (75.0-99.2) | 2,372 |  | 84.9 (74.0-96.4) | 565 |  | 92.9±17.1** | 273 |
| BMI, kg/m2 | 28.2 (25.2-32.2) | 2,373 |  | 27.8 (24.8-31.2) | 565 |  | 30.4 (26.6-33.8)** | 273 |
| HR, bmp | 64.0 (56.0-72.0) | 2,220 |  | 64.0 (57.0-72.0) | 558 |  | 65.9 (57.8-74.0) | 273 |
| Systolic BP, mmHg | 129.0 (92.9-218.0) | 2,143 |  | 127.6 (115.0-138.0) | 482 |  | 133.3±29.1** | 247 |
| Diastolic BP, mmHg | 77.0 (55.4-104.7) | 2,142 |  | 77.6 (69.0-85.0) | 481 |  | 77.6±14.6 | 247 |
| Sarcomere mutation positive | 823 (36) | 2,285 |  | 221 (41) | 541 |  | 68 (26)* | 260 |
| NYHA class |  | 2,344 |  |  | 554 |  |  | 267 |
| I | 1,562 (67) |  |  | 384 (69) |  |  | 135 (50)** |  |
| II | 618 (26) |  |  | 149 (27) |  |  | 108 (40)** |  |
| III/IV | 164 (7) |  |  | 21 (4) |  |  | 22 (8)** |  |
| Family history SCD |  | 2,366 |  |  | 561 |  |  | 270 |
| 1st degree | 295 (12) |  |  | 78 (14) |  |  | 29 (11) |  |
| 2nd degree | 289 (12) |  |  | 74 (13) |  |  | 32 (12) |  |
| Syncope | 322 (14) | 2,369 |  | 80 (14) | 562 |  | 37 (14) | 271 |
| NSVT | 172 (12) | 1,436 |  | 55 (15) | 379 |  | 22 (13) | 176 |
| Smoker | 306 (13) | 2,368 |  | 78 (14) | 561 |  | 32 (12) | 271 |
| AF | 270 (11) | 2,367 |  | 55 (10) | 562 |  | 23 (8) | 271 |
| Hypertension | 858 (36) | 2,369 |  | 171 (30) | 562 |  | 125 (46)** | 271 |
| Diabetes | 195 (8) | 2,369 |  | 31 (6) | 562 |  | 22 (8) | 271 |
| Medications |  |  |  |  |  |  |  |  |
| β-blockers | 1331 (56) | 2,362 |  | 287 (51) | 560 |  | 192 (71)** | 269 |
| CCB | 450 (17) | 2,362 |  | 92 (16) | 560 |  | 57 (21) | 269 |
| Disopyramide | 69 (3) | 2,362 |  | 4 (1) | 560 |  | 11 (4)** | 269 |
| Anticoagulant | 215 (9) | 2,362 |  | 49 (9) | 560 |  | 13 (5) | 269 |
| ACE/ARB | 572 (22) | 2,362 |  | 125 (22) | 560 |  | 62 (23) | 269 |
| Diuretics | 273 (10) | 2,362 |  | 51 (9) | 560 |  | 30 (11) | 269 |
| Amiodarone | 41 (2) | 2,362 |  | 6 (1) | 560 |  | 2 (1) | 269 |
| Statin | 369 (16) | 2,362 |  | 82 (15) | 560 |  | 59 (22) | 269 |

Values are given as mean±SD or median (25% - 75% percentiles) or n (% with respect to number of valid cases). From the total number of cases in the HCMR cohort, only those used for discriminant analysis are reported. **P-value<0.05 compared to R-S- group. HCM: Hypertrophic Cardiomyopathy; oHCM: Hypertrophic obstructive cardiomyopathy. BMI: Body mass index; HR: Heart rate; BP: Blood pressure; NYHA: New York Heart Association; SCD: Sudden cardiac death; NSVT: Non-sustained ventricular tachycardia; AF: Atrial fibrillation; CCB: Calcium channel blocker; ACE/ARB: Angiotensin-converting enzyme/angiotensin receptor blocker. HCMR group R-S- (HCM_R-S-_): HCM non-obstructive cases at rest and stress; HCMR group R-S+ (oHCM_R-S+_): HCM obstructive cases at stress but not at rest.

**Table 3. External Cohort - OCMR baseline characteristics.**

|  | **Total**  **(n = 101)** | **Valid** |  | **HCM_R-_**  **(n = 87)** | **Valid** |  | **OHCM_R+_**  **(n = 14)** | **Valid** |
| --- | --- | --- | --- | --- | --- | --- | --- | --- |
| Age, yrs | 52±14 | 99 |  | 51±14 | 85 |  | 55±12 | 14 |
| Male | 79 (80) | 99 |  | 71 (84) | 85 |  | 8 (57) | 14 |
| Weight, kg | 83.6±15.3 | 99 |  | 83.4±15.4 | 85 |  | 84.7±15.8 | 14 |
| BMI, kg/m2 | 27.7 (25.2-30.2) | 99 |  | 27.8 (25.1-30.1) | 85 |  | 28.0±4.1 | 14 |
| NYHA class |  | 90 |  |  | 78 |  |  | 12 |
| I | 72 (80) |  |  | 66 (85) |  |  | 6 (50)* |  |
| II | 13 (15) |  |  | 8 (10) |  |  | 5 (42)* |  |
| III/IV | 5 (5) |  |  | 4 (5) |  |  | 1 (8)* |  |
| Family history SCD |  | 90 |  |  | 78 |  |  | 12 |
| 1st degree | 9 (10) |  |  | 9 (12) |  |  | 0 (0) |  |
| 2nd degree | 9 (10) |  |  | 9 (12) |  |  | 0 (0) |  |
| Syncope | 13 (14) | 90 |  | 12 (15) | 78 |  | 1 (8) | 12 |
| NSVT | 11 (12) | 90 |  | 10 (13) | 78 |  | 1 (8) | 12 |
| Smoker | 8 (9) | 90 |  | 7 (9) | 78 |  | 1 (8) | 12 |
| AF | 4 (4) | 90 |  | 4 (5) | 78 |  | 0 (0) | 12 |
| Hypertension | 31 (34) | 90 |  | 26 (33) | 78 |  | 5 (42) | 12 |
| Diabetes | 8 (9) | 90 |  | 7 (9) | 78 |  | 1 (8) | 12 |
| Medications |  |  |  |  |  |  |  |  |
| β-blockers | 46 (51) | 90 |  | 37 (47) | 78 |  | 9 (75) | 12 |
| CCB | 22 (24) | 90 |  | 18 (23) | 78 |  | 4 (33) | 12 |
| Disopyramide | 9 (10) | 90 |  | 5 (6) | 78 |  | 4 (33)* | 12 |
| Anticoagulant | 1 (1) | 90 |  | 1 (1) | 78 |  | 0 (0) | 12 |
| ACE/ARB | 24 (27) | 90 |  | 21 (27) | 78 |  | 3 (25) | 12 |
| Diuretics | 0 (0) | 90 |  | 0 (0) | 78 |  | 0 (0) | 12 |
| Amiodarone | 7 (8) | 90 |  | 4 (5) | 78 |  | 3 (25) | 12 |
| Statin | 22 (24) | 90 |  | 19 (24) | 78 |  | 3 (25) | 12 |

Values are given as mean±SD or median (25% - 75% percentiles) or n (% with respect to number of valid cases). *P-value<0.05 compared to R- group. HCM: Hypertrophic Cardiomyopathy; oHCM: Obstructive hypertrophic cardiomyopathy. BMI: Body mass index; NYHA: New York Heart Association; SCD: Sudden cardiac death; NSVT: Non-sustained ventricular tachycardia; AF: Atrial fibrillation; CCB: Calcium channel blocker; ACE/ARB: Angiotensin-converting enzyme/angiotensin receptor blocker. OCMR group R- (HCM_R-_): HCM non-obstructive cases at rest; OCMR group R+ (oHCM_R+_): HCM obstructive cases at rest.

**Table 4. HCMR imaging characteristics – Groups R- and R+.**

|  | **Total**  **(n = 2,377)** | **Valid** |  | **HCM_R-_**  **(n = 1,320)** | **Valid** |  | **oHCM_R+_**  **(n = 438)** | **Valid** |
| --- | --- | --- | --- | --- | --- | --- | --- | --- |
| Mean LVWT, mm | 8.5 (7.4-10.0) | 2,377 |  | 8.7 (7.4-10.0) | 1,320 |  | 8.8 (7.5-9.9) | 438 |
| Max LVWT, mm | 16.9 (14.2-20.3) | 2,377 |  | 17.5 (14.4-20.7) | 1,320 |  | 17.5 (14.6-20.1) | 438 |
| LVEDV, mL | 169.1 (143.8-195.3) | 2,356 |  | 167.3 (141.6-196.9) | 1,308 |  | 175.1±42.5* | 434 |
| LVEDVI, mL/m2 | 84.2 (73.8-94.2) | 2,356 |  | 83.3 (73.1-93.7) | 1,308 |  | 86.4 (75.8-96.3)* | 434 |
| LVESV, mL | 60.9 (44.7-75.9) | 2,356 |  | 59.7 (44.6-74.1) | 1,308 |  | 62.9 (44.8-78.5) | 434 |
| LVESVI, mL/m2 | 30.1 (22.9-36.7) | 2,356 |  | 29.8 (22.7-36.2) | 1,308 |  | 30.9 (23.4-37.7) | 434 |
| LVSV, mL | 107.2 (94.3-121.9) | 2,356 |  | 106.3 (93.3-121.2) | 1,308 |  | 110.3 (96.0-125.0)* | 434 |
| LVSVI, mL/m2 | 53.7 (47.5-60.1) | 2,356 |  | 53.2 (47.2-59.9) | 1,308 |  | 55.0 (48.2-61.6)* | 434 |
| LVEF, % | 64.6 (59.2-70.2) | 2,356 |  | 64.7 (59.5-70.0) | 1,308 |  | 64.5 (58.8-70.5) | 434 |
| LVM, g | 165.1 (128.8-199.3) | 2,356 |  | 159.0 (125.6-190.4) | 1,308 |  | 171.7±58.6* | 434 |
| LVMI, g/m2 | 81.6 (65.5-96.7) | 2,356 |  | 78.9 (64.3-92.4) | 1,308 |  | 94.4 (74.3-115.3)* | 434 |
| LGE | 1,169 (50) | 2,349 |  | 627 (48) | 1,306 |  | 208 (48) | 431 |
| LVOT pressure drop rest, mmHg |  |  |  | 7.9 (3.0-12.0) | 1,320 |  | 64.1 (42.1-82.8)* | 438 |

Values are given as mean±SD or median (15% - 75% percentiles) or n (% with respect to number of valid cases). *P-value<0.05 compared to R-S- group. LVWT: Left ventricular wall thickness; LVEDV: Left ventricular end-diastolic volume; LVEDVI: Left ventricular end-diastolic volume indexed; LVESV: Left ventricular end-systolic volume; LVESVI: Left ventricular end-systolic volume indexed; LVSV: Left ventricular stroke volume; LVSVI: Left ventricular stroke volume indexed; LVEF: Left ventricular ejection fraction; LVM: Left ventricular mass; LVMI: Left ventricular mass index; LGE: presence of late gadolinium enhancement. LVOT: Left ventricular outflow tract. Mean and Max LVWT measures derived from the personalized 3-dimensional meshes. The rest of imaging characteristics were derived from the CMR images. HCMR group R- (HCM_R-_): HCM non-obstructive cases at rest; HCMR group R+ (oHCM_R+_): HCM obstructive cases at rest.

**Table 4. HCMR imaging characteristics – Groups R-S- and R-S+**

|  | **Total**  **(n = 2,377)** | **Valid** |  | **HCM_R-S-_**  **(n = 565)** | **Valid** |  | **OHCM_R-S+_**  **(n = 273)** | **Valid** |
| --- | --- | --- | --- | --- | --- | --- | --- | --- |
| Mean LVWT, mm | 8.5 (7.4-10.0) | 2,377 |  | 8.7 (7.3-10.1) | 565 |  | 8.9 (7.5-10.3) | 273 |
| Max LVWT, mm | 16.9 (14.2-20.3) | 2,377 |  | 17.4 (14.2-20.8) | 565 |  | 17.8 (14.7-20.7) | 273 |
| LVEDV, mL | 169.1 (143.8-195.3) | 2,356 |  | 169.3±39.9 | 558 |  | 176.5±42.5** | 273 |
| LVEDVI, mL/m2 | 84.2 (73.8-94.2) | 2,356 |  | 83.3 (48.9-93.4) | 558 |  | 86.9±16.7 | 273 |
| LVESV, mL | 60.9 (44.7-75.9) | 2,356 |  | 59.3 (44.5-74.1) | 558 |  | 59.9 (45.0-74.1) | 273 |
| LVESVI, mL/m2 | 30.1 (22.9-36.7) | 2,356 |  | 29.7 (23.0-36.0) | 558 |  | 28.9 (21.9-35.8) | 273 |
| LVSV, mL | 107.2 (94.3-121.9) | 2,356 |  | 105.3 (93.4-119.0) | 558 |  | 111.9±25.3** | 273 |
| LVSVI, mL/m2 | 53.7 (47.5-60.1) | 2,356 |  | 53.2 (47.3-59.8) | 558 |  | 53.7 (47.1-60.9) | 273 |
| LVEF, % | 64.6 (59.2-70.2) | 2,356 |  | 64.6 (59.3-69.8) | 558 |  | 65.7 (60.7-71.4) | 273 |
| LVM, g | 165.1 (128.8-199.3) | 2,356 |  | 156.9 (125.2-183.7) | 558 |  | 198.0±70.2** | 273 |
| LVMI, g/m2 | 81.6 (65.5-96.7) | 2,356 |  | 78.3 (63.9-91.3) | 558 |  | 97.1±30.0** | 273 |
| LGE | 1,169 (50) | 2,349 |  | 673 (52) | 558 |  | 190 (44) | 273 |
| LVOT pressure drop rest, mmHg |  |  |  | 5.6 (0.0-8.0) | 565 |  | 15.3 (9.9-21.0)** | 273 |
| LVOT pressure drop stress, mmHg |  |  |  | 8.6 (2.0-14.0) | 565 |  | 64.2 (42.0-85.0)** | 273 |

Values are given as mean±SD or median (25% - 75% percentiles) or n (% with respect to number of valid cases). **P-value<0.05 compared to R-S- group. LVWT: Left ventricular wall thickness; LVEDV: Left ventricular end-diastolic volume; LVEDVI: Left ventricular end-diastolic volume indexed; LVESV: Left ventricular end-systolic volume; LVESVI: Left ventricular end-systolic volume indexed; LVSV: Left ventricular stroke volume; LVSVI: Left ventricular stroke volume indexed; LVEF: Left ventricular ejection fraction; LVM: Left ventricular mass; LVMI: Left ventricular mass index; LGE: presence of late gadolinium enhancement. LVOT: Left ventricular outflow tract. Mean and Max LVWT measures derived from the personalized 3-dimensional meshes. The rest of imaging characteristics were derived from the CMR images. HCMR group R-S- (HCM_1_): HCM non-obstructive cases at rest; HCMR group R-S+ (oHCM_R-S+_): HCM obstructive cases at rest.

**Table 5. External cohort - OCMR imaging characteristics.**

|  | **Total**  **(n = 101)** | **Valid** |  | **HCM_R-_**  **(n = 87)** | **Valid** |  | **OHCM_R+_**  **(n = 14)** | **Valid** |
| --- | --- | --- | --- | --- | --- | --- | --- | --- |
| Mean LVWT, mm | 9.5±2.1 | 101 |  | 9.5±2.1 | 87 |  | 9.7±1.9 | 14 |
| Max LVWT, mm | 16.2±4.7 | 101 |  | 16.2±4.7 | 87 |  | 16.6±4.8 | 14 |
| LVEDV, mL | 144.7±38.1 | 90 |  | 142.2±38.4 | 78 |  | 160.4±33.1 | 12 |
| LVESV, mL | 40.6 (31.4-51.4) | 90 |  | 41.5±16.0 | 78 |  | 43.3±13.2 | 12 |
| LVSV, mL | 100.4 (83.7-118.5) | 90 |  | 100.7±27.5 | 78 |  | 117.1±24.9 | 12 |
| LVEF, % | 71.4±6.6 | 90 |  | 70.3 (67.0-74.7) | 78 |  | 75.2 (70.0-77.1) | 12 |
| LVM, g | 164.0 (135.9-186.3) | 90 |  | 160.2 (138.0-181.6) | 78 |  | 193.3±54.3 | 12 |
| LVMI, g/m2 | 80.7 (70.7-92.3) | 90 |  | 79.6 (70.2-90.3) | 78 |  | 94.7±22.9 | 12 |
| LGE | 73 (81) | 90 |  | 62 (79) | 78 |  | 11 (92) | 12 |
| LVOT pressure drop, mmHg |  |  |  | 10.8 (8.4-15.7) | 87 |  | 44.7±9.9* | 14 |

Values are given as mean±SD or median (25% - 75% percentiles) or n (% with respect to number of valid cases). **P-value<0.05 compared to R-group. LVWT: Left ventricular wall thickness; LVEDV: Left ventricular end-diastolic volume; LVESV: Left ventricular end-systolic volume; LVSV: Left ventricular stroke volume; LVEF: Left ventricular ejection fraction; LVM: Left ventricular mass; LVMI: Left ventricular mass index; LGE: presence of late gadolinium enhancement. LVOT: Left ventricular outflow tract. Mean and Max LVWT measures derived from the personalized 3-dimensional meshes. OCMR group R- (HCM_R-_): HCM non-obstructive cases at rest; OCMR group R+ (oHCM_R+_): HCM obstructive cases at rest.

**Figure 1. Study of the effect of changing the threshold of resting obstruction from 30 to 50 mmHg on the anatomical signature of the presence of resting LVOTO in the HCMR cohort.** Panel a) shows the overlay between the extremes of variation within our study population with the two thresholds (green: 50 mmHg; grey: 30 mmHg). Panel b) shows the corresponding Bull’s eye plots. The red point indicates the position of the right ventricle (septal wall) and the red arch the location of the left ventricular outflow tract. Apart from the subtle shape changes shown, discriminatory performance of the LDA_rest_ did not significantly change when using 30 or 50 mmHg, both in leave-one-out cross validation (L1) and resubstitution (RS): 0.760 RS and 0.742 L1 (30 mmHg); 0.763 RS and 0.739 L1 (50 mmHg).

**Figure 2. Differences in the LDA_rest_** **when using paired cases from the LDA_∆stress_ model.** Panel a) shows the reference LDA_rest_ model built with all available cases with information about their resting obstructive status. Panel b) shows the extra LDA_rest_ model built using paired cases from the LDA_∆stress_. The extra model showed the same qualitative characteristics as the original LDA_rest_ model, but with milder thickening patterns in the obstructive extreme shape (i.e., +3SD from the average shape).

**Figure 3. Study of the effect of β-blockers on the discriminant axis at rest (LDArest).** The distribution of Z- scores along the axis of anatomical variation that best predicts the presence of resting LVOTO is shown for each subgroup: non-obstructive HCM without β-blockers (light blue); HCM on β-blockers (green); obstructive HCM (oHCM) without β-blockers (red); oHCM on β-blockers. Orange cross and 3D shapes show -3 SD from the mean shape (blue cross); purple cross and shape, +3 SD. Red arch: LVOT location is clarified with a red arch (note Bull’s eye plot (bottom) is viewed from the apex, model views are from lateral (top-left) and base (top-right)). Red dot: septal wall location. A: Anterior; I: Inferior; L: Lateral; S: Septal.
