# Supplementary figures and images for: Left ventricular anatomy in obstructive hypertrophic cardiomyopathy: beyond basal septal hypertrophy
Source: Eur Heart J Cardiovasc Imaging. 2022 Nov 28;24(6):807–18. doi: 10.1093/ehjci/jeac233 (PMC10229266; doi:10.1093/ehjci/jeac233)

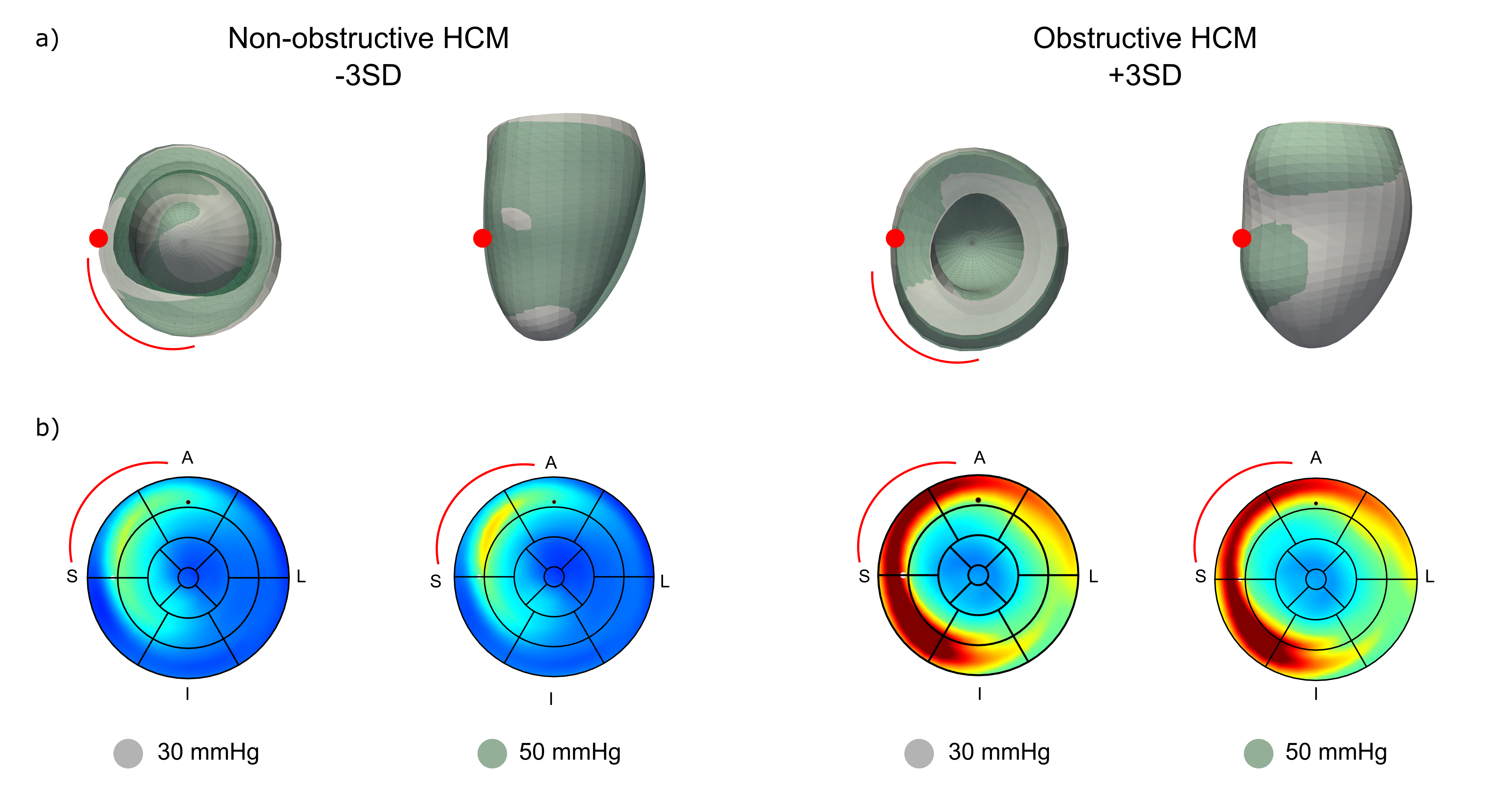

Supplement: jeac233_Supplementary_Data [file jeac233_supplementary_data.zip › Supplementary Figure 1.png]

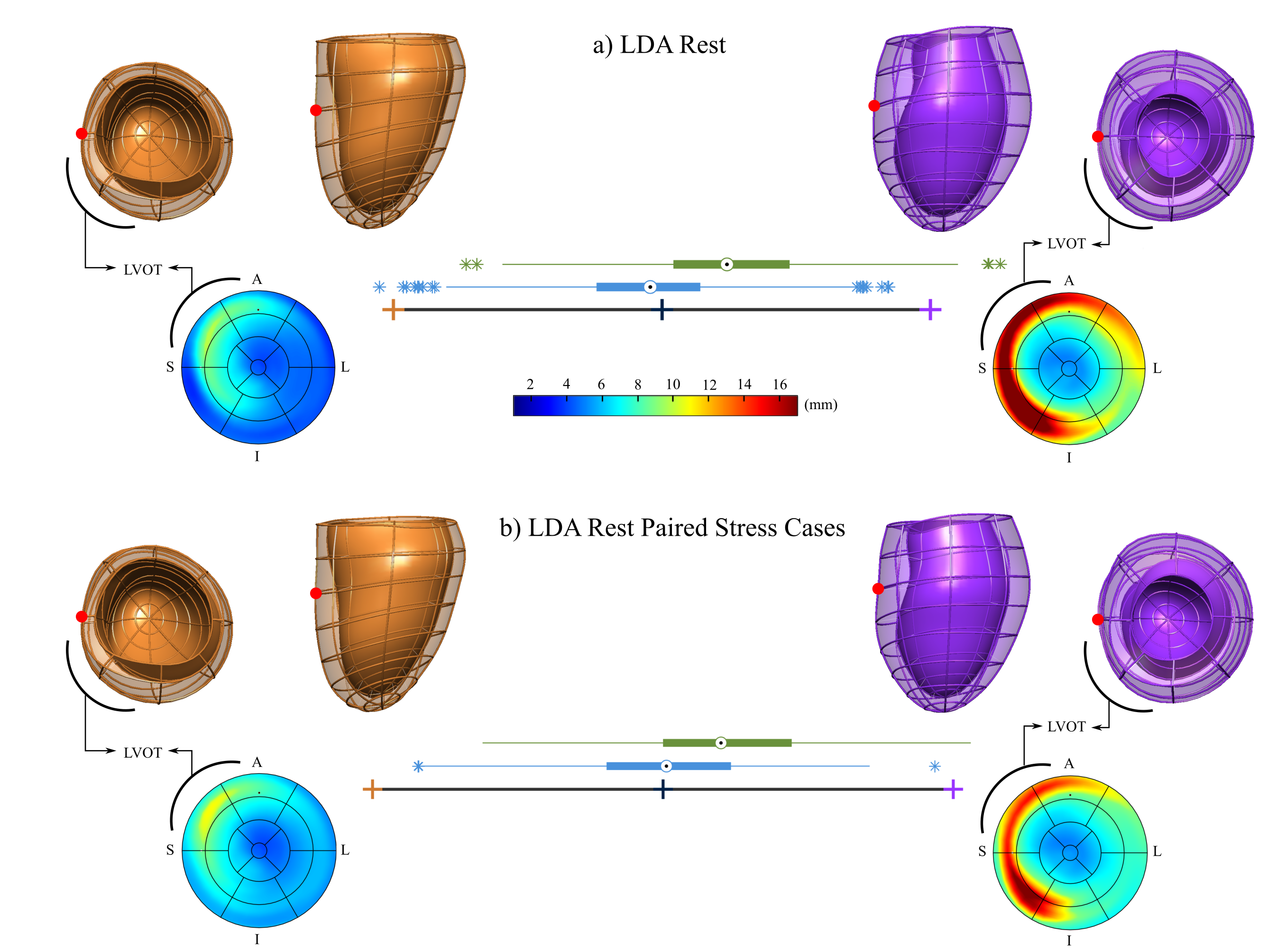

Supplement: jeac233_Supplementary_Data [file jeac233_supplementary_data.zip › Supplementary Figure 2.png]

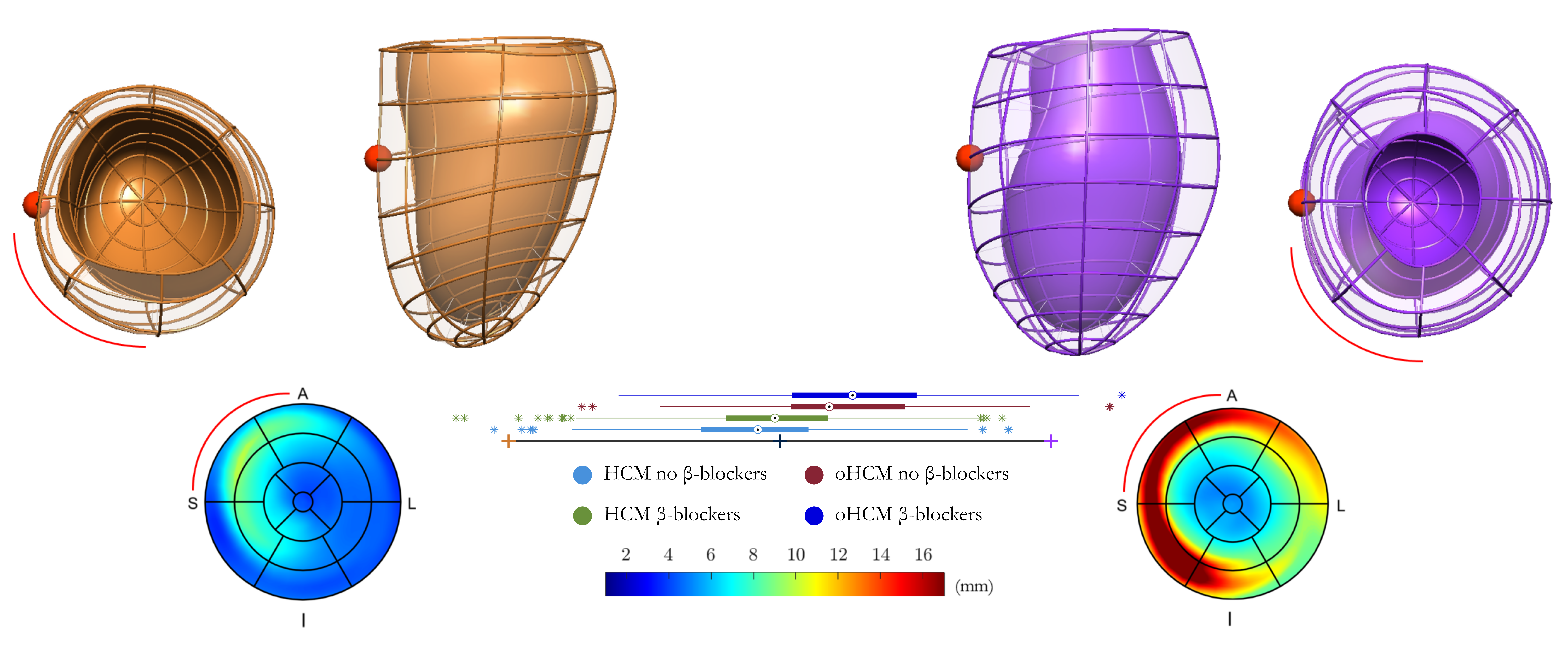

Supplement: jeac233_Supplementary_Data [file jeac233_supplementary_data.zip › Supplementary Figure 3 (1).png]
